# Ablation Zone Involution of Liver Tumors Is Faster in Patients Treated with Irreversible Electroporation Than Microwave Ablation

**DOI:** 10.3390/medicina57090877

**Published:** 2021-08-26

**Authors:** Fourat Ridouani, Mario Ghosn, Francois Cornelis, Elena N Petre, Meier Hsu, Chaya S Moskowitz, Peter T Kingham, Stephen B Solomon, Govindarajan Srimathveeravalli

**Affiliations:** 1 Interventional Radiology Service, Memorial Sloan Kettering Cancer Center, New York, NY 10065, USA; ridouanf@mskcc.org (F.R.); ghosnm@mskcc.org (M.G.); petree@mskcc.org (E.N.P.); solomons@mskcc.org (S.B.S.); 2 Department of Interventional Radiology and Oncology, Tenon Hospital, Sorbonne University, 4 Rue de la Chine, 75020 Paris, France; cornelisfrancois@gmail.com; 3 Biostatistics Service, Department of Epidemiology and Biostatistics, Memorial Sloan Kettering Cancer Center, New York, NY 10065, USA; hsum1@mskcc.org (M.H.); moskowc1@mskcc.org (C.S.M.); 4 HepatoPancreatoBiliary Service, Department of Surgery, Memorial Sloan Kettering Cancer Center, New York, NY 10065, USA; kinghamP@mskcc.org; 5 Department of Mechanical & Industrial Engineering, University of Massachusetts, Amherst, MA 01002, USA; 6 Institute for Applied Life Sciences, University of Massachusetts, Amherst, MA 01002, USA

**Keywords:** liver, regeneration, hepatocellular carcinoma, metastasis, microwave ablation, irreversible electroporation

## Abstract

*Background and Objectives*: To compare ablation zone involution following microwave ablation (MWA) or irreversible electroporation (IRE) of liver tumors. *Materials and Methods*: MWA or IRE performed for colorectal cancer liver metastasis (CRLM) or hepatocellular carcinoma (HCC) during January 2011 to December 2015 were analyzed. Patients with a tumoral response on 1-year follow-up computed tomography (CT) were included. Generalized estimating equations were used to evaluate the differences between the two modalities on ablation zone involution observed on CT at 6 (M6) and 12 months (M12), and on laboratory values (total bilirubin, alanine transaminase, aspartate transaminase, alkaline phosphatase, albumin, and platelets count). The likelihood ratio test was used to assess whether the association between ablation modalities and these outcomes differed over time. *Results*: Seventeen (17/44, 39%) women and 27 (27/44, 61%) men were included, with 25 HCC (25/44, 57%) and 19 CRLM (19/44, 43%) patients. IRE was used in 9 (9/19, 47%) CRLM and 5 (5/25, 20%) HCC patients, respectively. All other patients had MWA. Ablation zone size and involution between IRE and MWA differed significantly over time (interaction *p* < 0.01), with a mean of 241.04 vs. 771.08 mm^2^ (ratio 0.313; 95% CI, 0.165–0.592; *p* < 0.01) at M6 and 60.47 vs. 589.43 mm^2^ (ratio 0.103; 95% CI, 0.029–0.365; *p* < 0.01) at M12. Changes in liver enzymes did not differ significantly between IRE and MWA at both timepoints. *Conclusions*: Liver tumors treated with IRE underwent faster involution when compared to tumors treated with MWA, but liver enzymes levels were comparable.

## 1. Introduction

Thermal ablation is performed for the treatment of primary or metastatic liver tumors by localized deposition of electromagnetic radiofrequency (radiofrequency ablation, RFA) [1,2,3,4] or microwave energy (microwave ablation, MWA) [5,6]. Thermal ablation has been shown to be safe and efficacious for liver tumors that are 3 cm or smaller [7,8,9,10,11,12] but is generally not recommended for tumors that abut or involve the bile duct or hepatic blood vessels, as it can compromise treatment safety and efficacy [13,14,15]. Irreversible electroporation (IRE) uses high voltage electric pulses to kill cells through disruption of the plasma membrane without sustained elevation of tissue temperature [16] and, therefore, has been evaluated for the treatment of tumors that are not amenable to thermal ablation [17,18,19,20]. IRE has been shown to be safe for tumors involving the bile duct or large blood vessels, with good clinical outcomes [16,17,21,22]. Sparing of the extracellular matrix and large blood vessels at the site of treatment is also an important difference between IRE and thermal ablation techniques [17,21,22,23].

Prior preclinical work by Bulvik et al. comparing thermal ablation and IRE of liver tumors in mice demonstrated differences in the temporal and spatial dynamics of the infiltration and activity of macrophages and other wound healing cells within the ablated tumor, with increased levels of penetration of such cells, and associated cytokine levels in IRE treated tumors [24]. Similarly, studies by Golberg et al. [25] and Li et al. [26] evaluating post-ablation regeneration of normal liver identified accelerated regeneration of treated liver following IRE but not thermal ablation. These findings have been confirmed by clinical observations by Sugimoto et al. [27], who noted significant early increases in macrophage migration inhibitory factor levels following IRE, which may facilitate the early reparative process. In contrast, no change was observed after RFA. A small single-arm study of six patients reported almost complete involution of the ablated tumors within one year following IRE [28]. 

Although the exact mechanisms underlying these observations have not been defined, preservation of the extracellular matrix and blood vessels at the site of IRE may be supportive of post-ablation liver recovery. Faster resolution of ablated tumor with subsequent liver regeneration following IRE may be beneficial for patients, providing additional rationale for exploring this technique for the ablation of liver tumors. The objective of our study was to discern differences in the rate of ablation zone involution following MWA or IRE of liver tumors in patients, using it as a proxy metric for localized liver healing and regeneration. Further, we sought to understand the influence of background liver parenchyma status on ablation zone involution by comparing imaging findings in patients with hepatocellular carcinoma (HCC) and metastatic colorectal cancer (CRLM).

## 2. Materials and Methods 

### 2.1. Study Design

This retrospective study collected data from patients who had computed tomography (CT) or ultrasound guided liver ablation with MWA or IRE from 2011 to 2015. Informed consent was waived for this health insurance portability and accountability act-compliant, institutional review board-approved study. 

### 2.2. Patient Population

Forty-four patients who had stable disease, complete or partial response according to modified response evaluation criteria in solid tumors on 1-year follow-up were included in this study [29]. Only responders were included to avoid any confounding effect of disease response to systemic therapy and ensure that the evolution of the ablation zone surface can be compared among patients. Only the first tumor treated with ablation was included for analysis in patients who underwent ablation of more than one tumor. Ablations using MWA or IRE guided with CT/Fluoroscopy or ultrasound imaging were performed for colorectal cancer liver metastasis (CRLM) or hepatocellular carcinoma (HCC). All cases were discussed at a multidisciplinary tumor board and ablation was indicated for cases deemed surgically unresectable or as a bridge to transplantation for the HCC patients. Decision regarding the ablation technique was determined based on the anatomic tumor location; IRE was performed on centrally located tumors in proximity to major vascular and biliary structures. Procedures were performed percutaneously or via laparotomy per clinical management strategy determined by the disease management team.

### 2.3. Ablation Techniques

All procedures were performed under general anesthesia. All ablations were performed in a single session. CT (with CT fluoroscopy capabilities) was used for real-time guidance during ablation probe placement and ablation monitoring; ultrasound guidance was used for ablation performed during surgical treatment.

MWA was performed using either NeuWave (NeuWave Medical, Madison, WI, USA), Amica (Mermaid Medical, Centennial, CO, USA), or Emprint (Medtronic, Minneapolis, MN, USA) systems. Ablation parameters were chosen at the discretion of the operator and according to the manufacturer’s recommendations, with the aim of achieving an ablation margin larger than 5 mm surrounding the tumor. Immediate post-ablation assessment of technical success was performed with either contrast-enhanced CT scan or ultrasound. If necessary, the probe placement was adjusted, and energy delivery was repeated to obtain the desired margin. 

All IRE ablations were performed using the Nanoknife system (AngioDynamics, Queensbury, NY, USA) in accordance to the manufacturer’s guidance. Applied voltage and other treatment parameters were adjusted using the manufacturer’s treatment planning tool (electrode exposure: 0.5–4 cm; pulse number: 70 to 90). Synchronization of the electrical pulses with the cardiac rhythm was performed in order to prevent arrhythmias. IRE patients underwent neuromuscular blockade to minimize muscle contraction during electric pulse application. 

For both IRE and MWA, technical success was defined per Ahmed et al. as completion of the treatment protocol with complete coverage of the tumor [3].

### 2.4. Imaging and Analysis

All patients had follow-up imaging (contrast enhanced CT-scan) immediately following the procedure, and 4 to 6 weeks post-ablation to assess technical efficacy. Subsequently, follow-up imaging consisted of a CT scan at 6 months and 12 months. The ablation zone was defined as the non-enhancing area of liver parenchyma on portal venousphase CT, as previously described for thermal ablations of hepatic malignancies [30]. The 2 largest axial diameters were recorded on portal venous-phase images and area was calculated to assess the shrinkage of the ablation zone. Involution was defined as a decrease in the surface area of the ablation zone. Normalized residual area was calculated and compared between the groups. 

Tumor liver segment was determined according to Couinaud classification. Tumors were classified as subcapsular if a distance of 5 mm or less separated them for the hepatic capsule, as previously defined by Kei et al. [31]. Presence of a vessel with >3 mm diameter within 5 mm of the ablation margin was also noted [31]. Each of these factors was compared between IRE and MWA treatment groups.

### 2.5. Liver Function Values

Cirrhosis status was identified according to the patients’ medical records, based on a combination of two or more of the following: patient’s history, clinical course, imaging findings, and liver histology results. All patients had a baseline laboratory assay obtained within 4 weeks prior to the procedure, at 24–48 h after the procedure and at each follow-up timepoint (6 and 12 months). Liver function tests (LFT), albumin and platelets values were recorded and compared chronologically before and after treatment.

### 2.6. Statistical Analysis

Distribution of continuous variables was summarized with means ± standard deviations (SD) and ranges. Categorical variables were presented as raw numbers, proportions, and percentages. Comparisons between groups were evaluated using the Wilcoxon rank sum test or Fisher’s exact test for continuous and categorical variables, respectively. The differences between ablation modalities on surface area shrinkage and laboratory values post-ablation were evaluated using generalized estimating equations to account for the correlation due to longitudinal measurements on the same patient. An independence covariance matrix was assumed. A natural logarithmic transformation was applied to normalize the distribution of skewed data. To evaluate whether the association between ablation modalities and ablation zone outcomes (surface area, total bilirubin (Bili), alanine transaminase (ALT), aspartate transaminase (AST), alkaline phosphatase (ALK), albumin (Alb) and platelets count (Plat)) differed over time, we assessed the significance of the interaction term for ablation type and time (as 3 categories) using the likelihood ratio test. The means were estimated from a model that modeled each outcome as a function of time, ablation modality, and the interaction of ablation modality and time. A similar approach was used to evaluate whether the interaction between ablation modality and surface area shrinkage differed by disease type (HCC vs. CRLM), by evaluating the significance of the 3-way interaction term for ablation modality, time, and disease type. All statistical analyses were performed using SAS 9.4 (SAS Institute, Cary, NC, USA) or R version 3.5.1 (R Foundation for Statistical Computing, Vienna, Austria). All tests were two-sided, and *p* values less than 0.05 were considered statistically significant.

## 3. Results

### 3.1. Population and Ablation Characteristics

A total of 44 patients were found eligible for inclusion in the study, with 17 (17/44, 39%) women and 27 (27/44, 61%) men. Patients underwent 30 (30/44, 68%) MWA ablations and 14 (14/44, 32%) IRE ablations for 25 (25/44, 57%) HCC and 19 (19/44, 43%) CRLM. The mean age of the patients was 61 ± 13 (SD) years (range: 44 to 82 years) for the IRE group and 67 ± 10 (SD) years (range: 43 to 81 years) for the MWA group. Mean tumor diameter was 17 ± 7 (SD) mm (range: 6 to 29 mm) in the IRE group and 21 ± 10 (SD) mm (range: 9 to 50 mm). Twenty-seven (27/44, 61%) patients were previously treated with systemic chemotherapy, but none had previous radiation therapy to the liver. Technical success was achieved in all cases.

For the HCC group, 5 (5/25, 20%) ablations were performed with IRE and 20 (20/25, 80%) were performed with MWA. In this group, the mean tumor largest diameter was 23 ± 6 (SD) mm (range: 13 to 29 mm) for the IRE ablations and 21 ± 11 (SD) mm (range: 9 to 50 mm) for the MWA ones. All patients with HCC who underwent IRE ablation had cirrhosis while 16 (16/20, 80%) in the MWA group had cirrhosis. 

For the CRLM group, 9 (9/19, 47%) patients had IRE ablation and 10 (10/19, 53%) had MWA ablation. The mean tumor largest diameter was 14 ± 5 (SD) mm (range: 6 to 25 mm) for the IRE and 20 ± 8 (SD) mm (range: 13 to 37 mm) for the MWA. None of the patients in the CRLM group had cirrhosis.

MWA was performed with a median power of 60 W (range: 30 to 100 W), for a median of 6 min (range: 2 to 10 min) and using a median of 1 applicator (range: 1 to 3). Applicators were repositioned in 14 cases (14/30, 47%). IRE was performed with a median of 2200 V/cm (range: 1500 to 3000 V/cm) for a median of 3 cycles (range: 3 to 4 cycles) and using a median of 2 applicators (range: 2 to 5 applicators). Applicators were repositioned in 1 cases (1/14, 7%).

Data for population characteristics, tumor size, subcapsular location, proximity with a >3 mm diameter vessel, prior systemic chemotherapy, and the baseline laboratory values for each group were comparable except for the location of tumors within the liver segments (Table 1).

### 3.2. Ablation Zone Involution

The ablation zone area immediately post procedure did not significantly differ between patients treated with IRE or MWA, with a fitted mean of 923.61 vs. 1136.03 mm^2^ (Ratio 0.813; 95% CI, 0.523–1.265; *p* = 0.36). The ablation zone area measured at 6 and 12 months timepoints differed significantly between the IRE and MWA groups, with a fitted mean of, respectively, 241.04 vs. 771.08 mm^2^ (Ratio 0.313; 95% CI, 0.165–0.592; *p* < 0.01) at 6 months and 60.47 vs. 589.43 mm^2^ (Ratio 0.103; 95% CI, 0.029–0.365; *p* < 0.01) at 12 months. The evolution of ablation zone area between IRE and MWA varied significantly over time (Interaction *p* < 0.01). Data are shown in Table 2.

### 3.3. Ablation Zone by Disease Type

**Figure 1:**
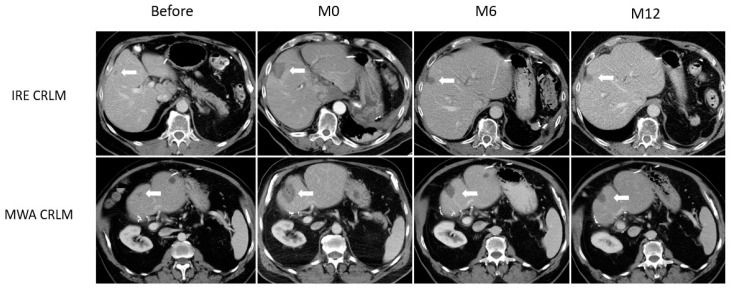
Example of ablation zone involution for CRLM. Axial CT slices showing 2 CRLM lesions (white arrow), both in segment IV, in a patient treated with IRE (upper row) and a patient treated with MWA (lower row). Lesions are shown before ablation, immediately after (M0), and at 6 (M6) and 12 months (M12) follow-up. In the CRLM subgroup, ablation surface area was significantly smaller at 6 and 12 months after IRE compared to MWA (Interaction *p* < 0.01).

In the CRLM subgroup, ablation surface area was significantly smaller at 6 and 12 months in IRE compared to MWA treated patients (Interaction *p* < 0.01), as illustrated in Figure 1.

**Figure 2:**
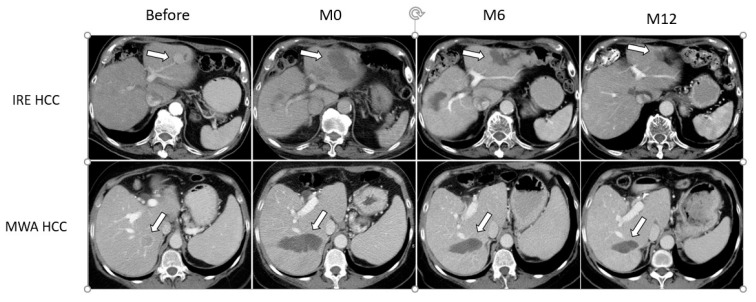
Example of ablation zone involution for HCC. Axial CT slices showing 2 HCC lesions (white arrow) in two different patients: one in left hemiliver (upper row) that was treated with IRE, and one in segments 6/7 (lower row) treated with MWA. Lesions are shown before ablation, immediately after (M0), and at 6 (M6) and 12 months (M12) follow-up. Although a perceptible difference with smaller ablation surface area is seen in patient treated with IRE, the difference between IRE and MWA did not reach statistical significance (Interaction *p* = 0.17).

In the HCC subgroup, although Figure 2 shows a perceptible difference with smaller ablation surface area in IRE treated patients, the difference between IRE and MWA was not statistically significant (Interaction *p* = 0.17). There was no statistical evidence to suggest there is a difference across disease types (*p* = 0.14). However, the power to detect a significant 3-way interaction is limited by the sample size. Results are shown in Table 3.

### 3.4. Liver Function

There was no significant difference in baseline laboratory values between IRE and MWA (Table 1). There was a median increase in bilirubin of 0.3 mg/dL (range: 0 to 1.4 mg/dL) 24–48 h post IRE and 0.2 mg/dL (range: −0.3 to 1.6 mg/dL) 24–48 h post MWA. AST and ALT increased after most procedures. ALK did not show the same patterns as transaminases and peak elevation was only noted in 10 (10/44, 23%) patients. Median increase in AST and ALT were 310.0 U/L (range: 18 to 1273 U/L) and 264.5 U/L (range: 49 to 1339 U/L) 24–48 h post IRE and 136 U/L (range: 11 to 579 U/L) and 101 U/L (range: 8 to 581 U/L) 24–48 h post MWA. There was a median decrease in albumin of 0.60 g/dL (range: −0.60 to 1.1 g/dL) for IRE and 0.3 g/dL (range: −0.40 to 0.8 g/dL) for MWA 24–48 h post procedure. The platelets count also decreased 24–48 h post procedure with a median drop of 46 K/mcL (range: −47 to 117 K/mcL) after IRE and 26 K/mcL (range: −84 to 112 K/mcL) after MWA.

**Figure 3:**
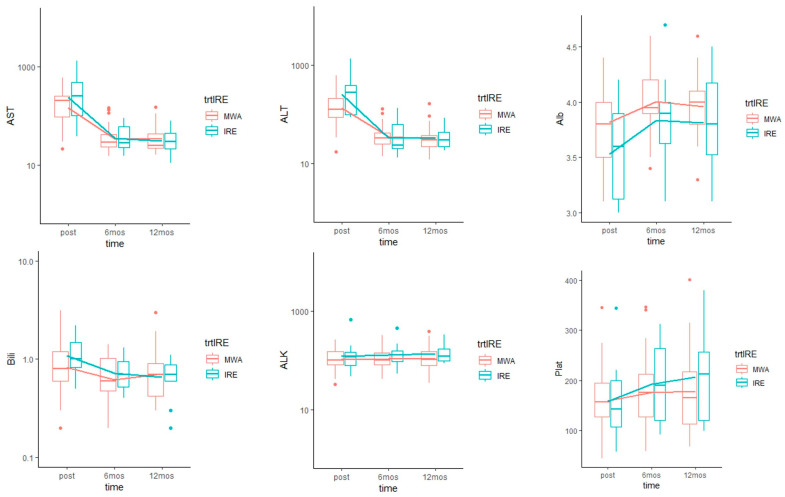
Evolution of laboratory values over time by IRE vs. MWA group. Boxplots with overlaid line graph of means of different laboratory values at each time point. Immediately after procedures, there was an increase in total bilirubin (Bili, Normal (N) < 1.2 mg/dL), alanine transaminase (ALT, N < 33 U/L), aspartate transaminase (AST, N < 37 U/L), and a decrease in albumin (Alb, N: 3.8–5 g/dL) and platelets count (Plat, N: 160–400 K/mcL). Alkaline phosphatase (ALK, N < 120 U/L) remained stable. All values returned to baseline by the 6- and 12-months follow-up in both cohorts. The trajectory of all of the laboratory values did not differ significantly overtime between IRE and MWA.

Bilirubin levels on average across all time points were not statistically significantly different between IRE and MWA (Ratio: 1.13, *p* = 0.36). Transaminases values immediately after IRE were slightly higher than after MWA, but AST (Ratio: 1.18, *p* = 0.28) and ALT (Ratio: 1.22, *p* = 0.25) levels on average across all time points were not statistically significantly different. Similarly, ALK (Ratio: 1.21, *p* = 0.2), platelet levels (Mean difference: 14.73, *p* = 0.52) were not statistically significantly different between IRE and MWA across all time points. Albumin levels were significantly lower for IRE than MWA, on average across all time points (Mean difference: −0.2, *p* = 0.03). Ultimately, all clinical lab values returned to baseline by the 6- and 12-months follow-up in both cohorts. The evolution of the laboratory values did not differ significantly overtime between IRE and MWA (Figure 3).

## 4. Discussion

This study showed that patients undergoing IRE of liver tumors demonstrated rapid involution of the ablation zone, at a faster rate than patients treated with MWA. These findings were observed at both the 6- and 12-month follow up timepoints, and were significant in patients with CRLM, but not in patients with HCC. Faster involution of the ablation zone in patients treated with IRE was not seen to impact laboratory values at follow up timepoints, which were similar to patients treated with MWA.

Although the difference in cell killing action between IRE and MWA along with the sparing of the extracellular matrix and blood vessels at the site of treatment are factors that could theoretically underlie our observations, the exact mechanism was not established in this study. The choice of ablation modality has largely been guided by clinical safety and efficacy considerations while post-ablation recovery has not been a consideration. Preclinical works by Bulvik et al. [24] revealed that post-ablation liver regeneration can have off-target tumorigenic effects, and at this point it is unclear whether rapid resolution of the ablation is desirable or is possibly deleterious. However, given equal efficacy, it may be logical to choose a tool that results in a shorter healing for the patient. A considerable volume of normal liver parenchyma is ablated during these procedures, essentially to provide a necessary safety oncological margin [32,33]. Therefore, IRE may be a good option for patients with impaired liver function as it provides a faster reparative process and less damage to surrounding tissue [26,27].

In a dedicated study comparing radiofrequency ablation and IRE in a healthy swine model liver regeneration was faster after IRE [26]. These findings were confirmed by Scheck et al. when assessing volumetric changes after IRE and RFA in patients with primary and secondary liver tumors [34]. In our study, we reported a significant difference in the recovery process after CRLM ablation, and even if there was also a trend after HCC ablation, we did not detect a statistical significance. Two explanations may be suggested: (i) a small sample size in the HCC group, with a limited power to detect a significant 3-way interaction, and (ii) a slower recovery process in the settings of cirrhotic liver. Reparative process after IRE could involve several mechanisms. Decreased damage to the microvasculature after IRE has been reported [35,36]; such intact vessels can potentially allow a better penetration of reparative cells into the ablated tissue. More recently, IRE was associated with an early increase in macrophage migration inhibitory factor (MIF) [27], which is believed to exert a hepatoprotective effect in the ischemic ablation zone, essentially in the surrounding reversibly damaged hepatocytes [37]. MIF also has antifibrotic effects in the liver, mediated by an AMPK-mediated pathway which facilitates early wound healing by inhibiting the platelet-derived growth factor-induced proliferation and migration of isolated hepatic stellate cells, leading to scarless liver regeneration [38]. In a recent study by Fujimori et al., authors compared IRE to MWA in normal lung, and showed that IRE preserved the extracellular matrix and was associated with an increase in macrophages and T lymphocytes in the ablated tissue, whereas these changes were only seen in the peripheral inflammatory rim of MWA [39].

Another finding in this study is that alteration in LFT resulted in a peak of transaminases and total bilirubin, with a simultaneous drop of albumin and platelets levels. This alteration in serum values was transient and eventually reverted to baseline. Serum values after IRE were slightly higher for transaminases and total bilirubin immediately post procedure but were not found to be significantly different in comparison to MWA. Previous studies also described higher elevation of transaminases after IRE ablation when compared to RFA [40,41], and similar results have been reported after cryoablation [42]. The exact mechanism underlying this increase is not clear; it may be related to leakage of cytosolic contents from reversibly electroporated hepatocytes at the ablation margin [40]. Despite an early transient increase, bilirubin levels in all IRE treated patients remained within reference values at follow-up evaluation even when treated tumors were within 1 cm of the biliary tree. This confirms the safety of IRE for locations considered contraindicated for thermal modalities. The short elevation in total bilirubin could be caused by transient compression of bile ducts within the ablation zone following edema and increased extracellular pressure [43]. Interestingly, rapid involution of the ablation zone following IRE was not associated with improved liver function. Ablation volumes tend to be a very small fraction (<5%) of the total liver volume and it is possible that regeneration of such a small volume of liver does not cause a measurable change in serum values.

This study has several limitations. First, the retrospective nature of the study impacting the selection of patients and the inclusion of single tumors successfully treated and available time points of laboratory values. Second, a small sample size of HCC patients treated with IRE ablation were included. Our results need to be confirmed with a larger study, ideally a randomized clinical trial. Third, although the decision on which ablation technique was performed was determined based on anatomic location, the two modalities were not statistically significantly different with respect to having tumors with proximity to >3 mm vessels. However, we are unable to completely rule out the influence of anatomic location on ablation outcomes due to our small sample size. Future studies with a larger cohort of patients are necessary to further validate our preliminary findings.

## 5. Conclusions

Post ablation serum levels of liver enzymes are comparable between IRE and MWA, but IRE was associated with a faster ablation zone involution, which, in addition to potentially decreasing complications when performing procedures adjacent to critical structures, may be clinically beneficial for some patients with impaired liver function. The reparative process was more significant after CRLM ablation; further studies are needed to examine the differences in outcomes across different cancer types. Quicker involution may also present benefits and synergies with immunotherapy that merit further investigation.

## Figures and Tables

**Table 1:** Distribution of clinical characteristics by ablation type.

Variable	IRE	MWA	*p*
	No = 14	No = 30	
**Age at ablation, years**	61 ± 13 (44–82)	67 ± 10 (43–81)	0.13
**Sex**			>0.9
Female	5 (5/14, 36%)	12 (12/30, 40%)	
Male	9 (9/14, 64%)	18 (18/30, 60%)	
**BMI, kg/m^2^**	26 ± 5 (17–33)	29 ± 7 (17–42)	0.21
**Prior systemic chemotherapy**	11 (11/14, 79%)	16 (16/30, 53%)	0.18
**Subcapsular location**	10 (10/14, 71%)	16 (16/30, 53%)	0.33
**Proximity to > 3 mm vessel**	12 (12/14, 86%)	18 (18/30, 60%)	0.16
**Liver segment**			**0.041**
Segment 1	4 (4/14, 29%)	0 (0/30, 0%)	
Segment 2	1 (1/14, 7.1%)	4 (4/30, 13%)	
Segment 3	0 (0/14, 0%)	1 (1/30, 3.3%)	
Segment 4	4 (4/14, 29%)	5 (5/30, 17%)	
Segment 5	2 (2/14, 14%)	4 (4/30, 13%)	
Segment 6	0 (0/14, 0%)	7 (7/30, 23%)	
Segment 7	1 (1/14, 7.1%)	2 (2/30, 6.7%)	
Segment 8	2 (2/14, 14%)	7 (7/30, 23%)	
**Tumor size, mm**	17 ± 7 (6–29)	21 ± 10 (9–50)	0.29
**Disease type**			0.10
CRLM	9 (6/14, 64%)	10 (10/30, 33%)	
HCC	5 (5/14, 36%)	20 (20/30, 67%)	
**Pre-treatment Bilirubin, mg/dL**	0.74 ± 0.22 (0.40–1.20)	0.64 ± 0.33 (0.20–1.50)	0.10
**Pre-treatment AST, U/L**	35 ± 17 (18–73)	38 ± 25 (15–125)	0.76
**Pre-treatment ALT, U/L**	38 ± 22 (16–92)	39 ± 25 (14–111)	0.71
**Pre-treatment ALK, U/L**	118 ± 94 (55–430)	105 ± 47 (36–211)	>0.9
**Pre-treatment Albumin, g/dL**	4.02 ± 0.35 (3.30–4.50)	4.11 ± 0.34 (3.40–5.00)	0.52
**Pre-treatment Platelet, K/mcL**	201 ± 62 (104–297)	181 ± 68 (81–368)	0.27

Data are presented as means ± standard deviations (range) or raw numbers (proportions, %). *p* values are from Wilcoxon rank sum test or Fisher’s exact test for continuous and categorical variables, respectively. Bold variable highlights main variable names; bold *p* value highlights the statistically significant *p* values.

**Table 2:** Effect of treatment on surface area at each time point.

Ablation Type	Time (Months)	Mean (mm²)	Ratio of IRE vs. MWA	95% CI	*p* Interaction
IRE	0	923.61	0.813	0.523–1.265	**<0.01**
MWA	0	1136.03			
IRE	6	241.04	0.313	0.165–0.592	
MWA	6	771.08			
IRE	12	60.47	0.103	0.029–0.365	
MWA	12	589.43			

The fitted mean is the estimated mean of surface area from the model. At each time point, the ratio of IRE vs. MWA and 95% CI are shown. A ratio < 1 means that the surface area for IRE is less than MWA whereas ratio > 1 means that the surface area for IRE is greater than MWA. The difference in surface area recovery between IRE and MWA varied significantly over time (interaction *p* < 0.01). Bold highlights the statistically significant *p* values.

**Table 3:** Effect of treatment on surface area at each time point by disease type.

Disease	Ablation Type	Time (months)	Mean (mm²)	Ratio of IRE vs. MWA	95% CI	*p* Interaction
**HCC**	IRE	0	1499.33	1.326	0.549–3.206	0.17
	MWA	0	1130.49			
	IRE	6	562.29	0.714	0.316–1.612	
	MWA	6	787.63			
	IRE	12	327.91	0.525	0.178–1.547	
	MWA	12	624.47			
**CRLM**	IRE	0	705.65	0.615	0.42–0.9	**<0.01**
	MWA	0	1147.19			
	IRE	6	150.57	0.204	0.1–0.415	
	MWA	6	739.00			
	IRE	12	23.64	0.045	0.009–0.219	
	MWA	12	525.13			

Within the subgroup of HCC, the difference in surface area recovery between IRE and MWA did not differ significantly over time (interaction *p* = 0.17). Within the subgroup of CRLM, the difference in surface area recovery between IRE and MWA differed significantly over time (interaction *p* < 0.01). Bold highlights the statistically significant *p* values.

## Data Availability

The data of the study are present at the Interventional Radiology department at Memorial Sloan Kettering Cancer Center. The data will be available upon reasonable request.

## Abbreviations

Alb: albumin; ALK: alkaline phosphatase; ALT: alanine transaminase; AST: aspartate transaminase; Bili: total bilirubin; CRLM: colorectal cancer liver metastasis; CI: confidence interval; CT: computed tomography; HCC: hepatocellular carcinoma; IRE: irreversible electroporation; LFT: liver function tests; MIF: migration inhibitory factor; MWA: microwave ablation; Plat: platelets count; RFA: radiofrequency ablation; SD: standard deviation.
